# The Lst Sialyltransferase of Neisseria gonorrhoeae Can Transfer Keto-Deoxyoctanoate as the Terminal Sugar of Lipooligosaccharide: a Glyco-Achilles Heel That Provides a New Strategy for Vaccines to Prevent Gonorrhea

**DOI:** 10.1128/mBio.03666-20

**Published:** 2021-03-23

**Authors:** Freda E.-C. Jen, Margaret R. Ketterer, Evgeny A. Semchenko, Christopher J. Day, Kate L. Seib, Michael A. Apicella, Michael P. Jennings

**Affiliations:** a Griffith University, Institute for Glycobiology, Gold Coast, Queensland, Australia; b Department of Microbiology and Immunology and Department of Internal Medicine, The University of Iowa, Iowa City, Iowa, USA; University of Mississippi Medical Center

**Keywords:** *Neisseria gonorrhea*, lipooligosaccharide (LOS), keto-deoxyoctanoate (KDO), gonococcal vaccine, *N*-acetylneuraminic acid (Neu5Ac), sialic acid, Lst sialyltransferase

## Abstract

The lipooligosaccharide (LOS) of Neisseria gonorrhoeae plays key roles in pathogenesis and is composed of multiple possible glycoforms. These glycoforms are generated by the process of phase variation and by differences in the glycosyltransferase gene content of particular strains. LOS glycoforms of N. gonorrhoeae can be terminated with an *N*-acetylneuraminic acid (Neu5Ac), which imparts resistance to the bactericidal activity of serum. However, N. gonorrhoeae cannot synthesize the CMP-Neu5Ac required for LOS biosynthesis and must acquire it from the host. In contrast, Neisseria meningitidis can synthesize endogenous CMP-Neu5Ac, the donor molecule for Neu5Ac, which is a component of some meningococcal capsule structures. Both species have an almost identical LOS sialyltransferase, Lst, that transfers Neu5Ac from CMP-Neu5Ac to the terminus of LOS. Lst is homologous to the LsgB sialyltransferase of nontypeable Haemophilus influenzae (NTHi). Studies in NTHi have demonstrated that LsgB can transfer keto-deoxyoctanoate (KDO) from CMP-KDO to the terminus of LOS in place of Neu5Ac. Here, we show that Lst can also transfer KDO to LOS in place of Neu5Ac in both N. gonorrhoeae and N. meningitidis. Consistent with access to the pool of CMP-KDO in the cytoplasm, we present data indicating that Lst is localized in the cytoplasm. Lst has previously been reported to be localized on the outer membrane. We also demonstrate that KDO is expressed as a terminal LOS structure *in vivo* in samples from infected women and further show that the anti-KDO monoclonal antibody 6E4 can mediate opsonophagocytic killing of N. gonorrhoeae. Taken together, these studies indicate that KDO expressed on gonococcal LOS represents a new antigen for the development of vaccines against gonorrhea.

## Abstract (precis)

The emergence of multidrug-resistant N. gonorrhoeae strains that are resistant to available antimicrobials is a current health emergency, and no vaccine is available to prevent gonococcal infection. Lipooligosaccharide (LOS) is one of the major virulence factors of N. gonorrhoeae.

## INTRODUCTION

Neisseria gonorrhoeae is a host-adapted bacterial pathogen that causes the sexually transmitted disease gonorrhea. Gonococcal infections can be symptomatic or asymptomatic, with up to 80% of infections in females being asymptomatic (1, 2). Untreated gonorrhea in females can lead to pelvic inflammatory disease (3), adverse pregnancy outcomes, neonatal complications, or infertility (4). Moreover, it can increase the risk of acquiring and transmitting HIV (5). It is estimated that there are more than 106 million cases of gonorrhea worldwide each year (6), and the emergence of multidrug-resistant (MDR) strains of N. gonorrhoeae is a major public health problem (7, 8). Isolates with resistance to nearly every antibiotic used to treat gonorrhea have been identified (9–12). In the past century, only three potential gonococcal vaccines have been tested in clinical trials, and none of these vaccines were successful (13–15). As such, there is an urgent unmet need for novel approaches for therapeutic and vaccine development.

**FIG 1:**
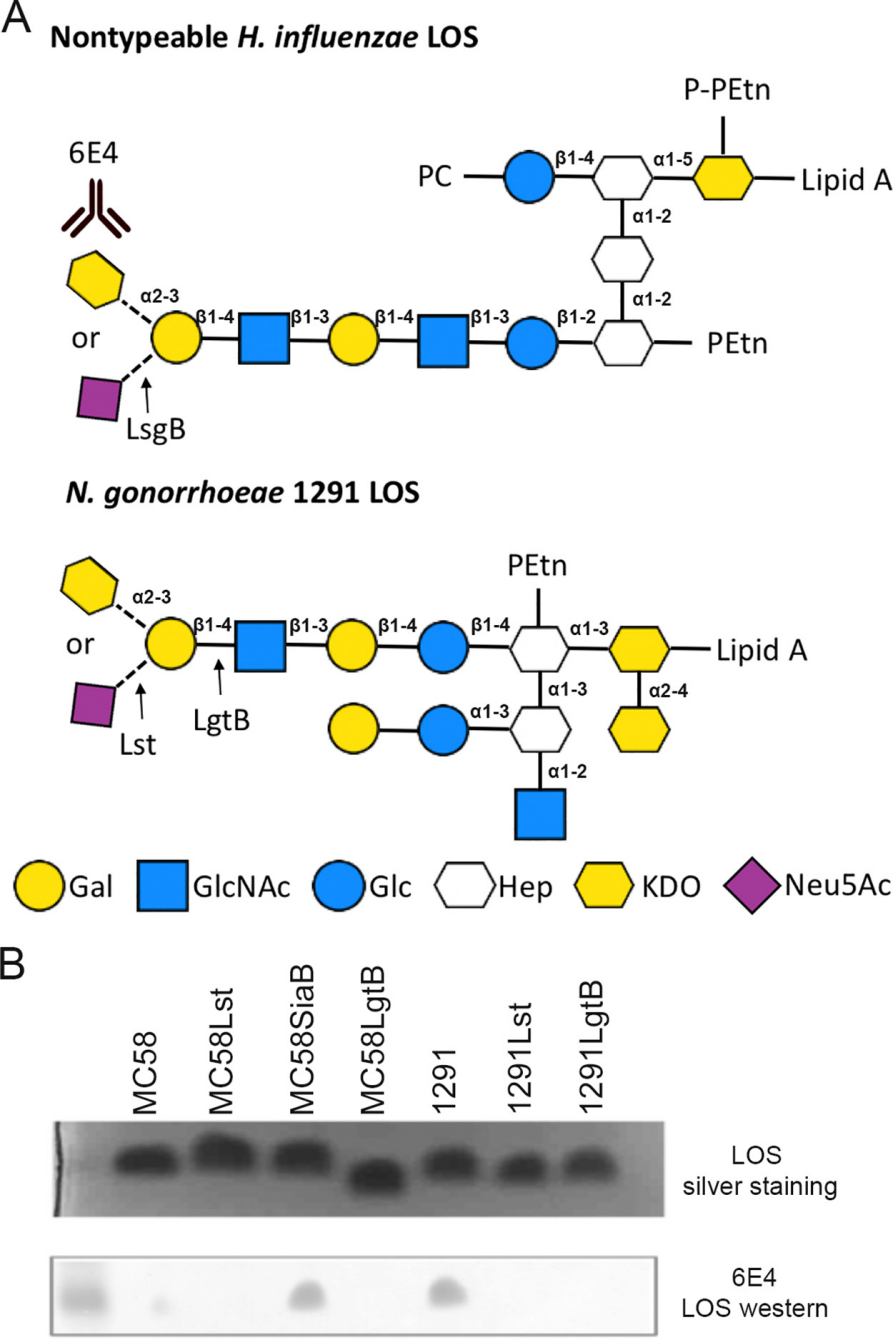
(A) Structure of the KDO-containing LOS of NTHi and N. gonorrhoeae. MAb 6E4 only recognizes the terminal KDO glycan. Lst and LsgB are the sialyltransferases that can also incorporate KDO onto the terminal Gal in the absence of Neu5Ac. LgtB is the galactosyltransferase that transfers the KDO acceptor galactose (Gal) to *N*-acetylglucosamine (GlcNAc). (B) Silver staining and 6E4 Western blot of purified LOS samples from MC58, 1291 wild-type, and glycotransferase mutant strains.

Lipooligosaccharide (LOS) is one of the major structural components of the outer membrane and is a key virulence factor of N. gonorrhoeae (16). It plays a number of key roles in the pathogenesis of N. gonorrhoeae, including mediating direct interaction between N. gonorrhoeae and human urethral epithelial cells (17, 18). The lipid A of LOS of N. gonorrhoeae binds to complement component C3, which is required for activation of complement receptor 3 in primary cervical epithelial cells (19). The sugar composition of LOS of N. gonorrhoeae is variable due to phase variation (high frequency of on/off switching of expression) of the genes involved in LOS biosynthesis (20–22). The LOS structure of N. gonorrhoeae can be terminated with an *N*-acetylneuraminic acid (Neu5Ac) (Fig. 1) (23–25), which has multiple roles in gonococcal virulence (reviewed in reference 26). However, N. gonorrhoeae cannot synthesize the nucleotide sugar precursor, cytidine-5′-monophosphate (CMP)-Neu5Ac, that is required for LOS biosynthesis and must acquire CMP-Neu5Ac from the host (24). In contrast, the closely related pathogen Neisseria meningitidis can synthesize CMP-Neu5Ac, which is also required for the polysaccharide capsule structure of meningococcal serogroups B, C, W, and Y (27). Both of these pathogenic *Neisseria* species have an almost identical (95% amino acid identity) sialyltransferase, Lst, that transfers Neu5Ac from CMP-Neu5Ac to the terminal galactose of LOS (Fig. 1A). The Lst sialyltransferase expressed by the pathogenic *Neisseria* species is homologous to the LsgB sialyltransferase of nontypeable Haemophilus influenzae (NTHi) (28, 29). Recent studies in NTHi have demonstrated that the LsgB sialyltransferase can transfer keto-deoxyoctanoate (KDO) from CMP-KDO to LOS in place of Neu5Ac (29) (Fig. 1A). Recent studies in N. gonorrhoeae have demonstrated that Lst can transfer non-Neu5Ac substrates to LOS (30, 31). In this study, we investigated whether KDO can also be incorporated as the terminal glycan on LOS of N. gonorrhoeae by Lst, which has significant implications for our understanding of sialic acid biology and gonococcal vaccine development.

## RESULTS

### The *Neisserial* Lst sialyltransferase can add KDO to gonococcal LOS.

**FIG 2:**
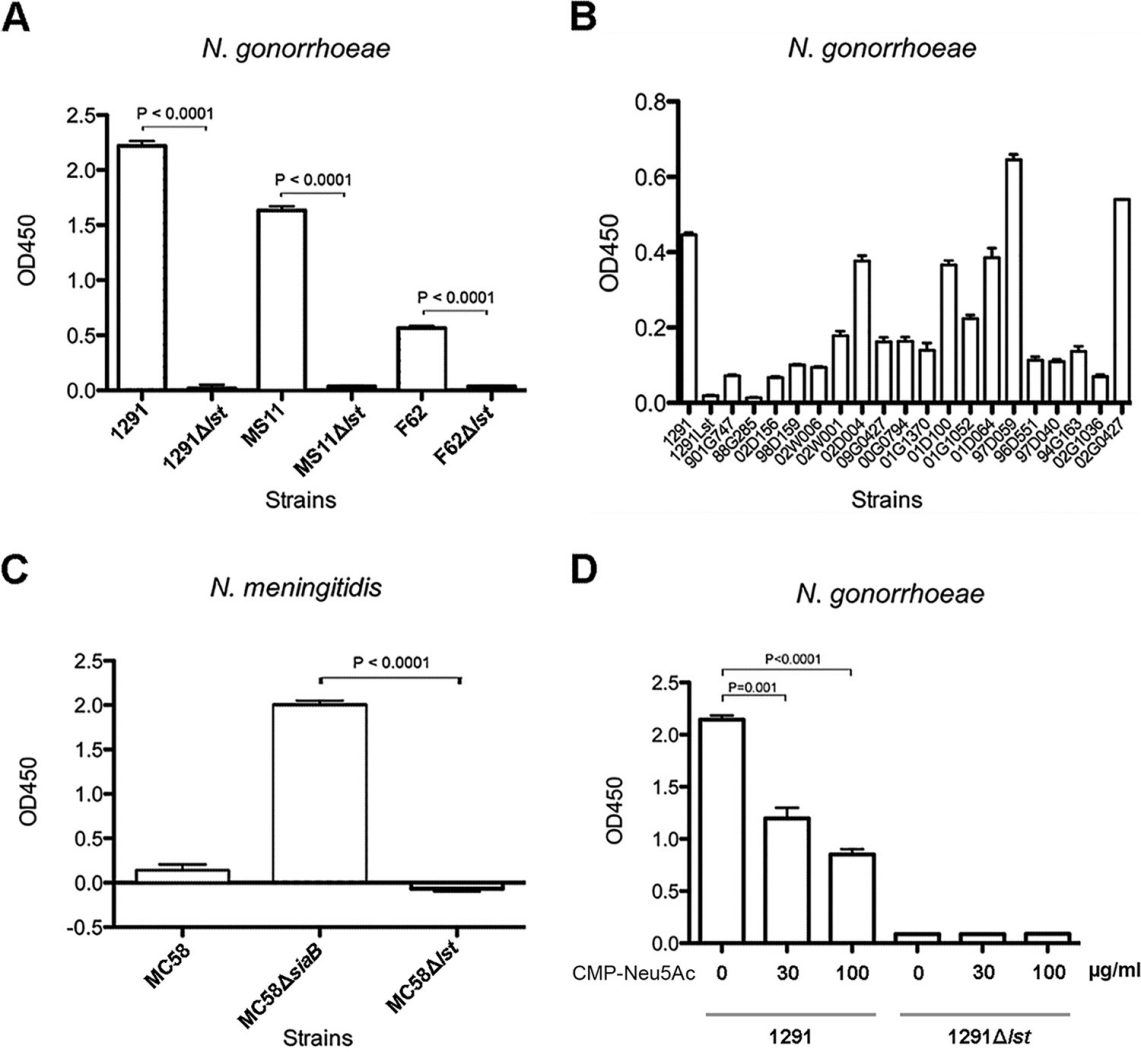
Whole-cell ELISA of 6E4. (A) ELISA of whole-cell N. gonorrhoeae WT and *lst* mutant strains using 6E4 MAb. Lst transfers the terminal sialic acid to the terminal Gal of LOS in N. meningitidis. Here, the *lst* mutant showed Lst also transfers KDO to the terminal Gal in N. gonorrhoeae. (B) Whole-cell ELISA of 6E4 was performed to test 20 clinical *N. gonorrhoeae* isolates. 1291 and 1291Lst were the positive and negative controls in this ELISA. (C) ELISA of whole-cell *Neisseria meningitidis* MC58 WT, SiaB, and *lst* mutant strains using 6E4 MAb. MC58 WT has Neu5Ac as the terminal glycan of LOS. Without SiaB, *N. meningitidis* cannot make CMP-Neu5Ac; therefore, Lst adds KDO at the terminal of LOS instead of sialic acid. (D) Incorporation of KDO on gonococcal LOS. Whole-cell ELISA of 6E4 showed that the presence of CMP-Neu5Ac in the growing medium could inhibit the incorporation of KDO into the terminal Gal. N. gonorrhoeae 1291 and *lst* mutant were grown in 0, 30 mM, and 100 μg/ml CMP-Neu5Ac supplemented media.

NTHi has four sialytransferases (SiaA, LsgB, Lic3A, and Lic3B) (32, 33), while N. gonorrhoeae has only one sialyltransferase, Lst (34). BLAST analysis indicated that Lst belongs to the glycosyltransferase superfamily 52 and is homologous to the sialyltransferase LsgB in NTHi (27% identity at the amino acid level, e value of 6e^−31^). To determine whether N. gonorrhoeae can transfer KDO as the terminal monosaccharide of LOS (29), similar to NTHi, whole-cell enzyme-linked immunosorbent assay (ELISA) was performed with the LOS terminal KDO-specific monoclonal antibody (MAb) 6E4 (29) on three well-characterized N. gonorrhoeae strains, 1291, MS11, and F62, and their *lst* knockout mutants. All three wild-type strains were KDO positive, while none of the *lst* mutants of these strains reacted with MAb 6E4 (Fig. 2A), indicating that Lst is required for KDO transfer to LOS. Consistent with previous studies, 6E4 cannot detect the basal KDO residue that is common to all LOS structures (29). Further analysis detected KDO (Fig. 2B) in 19 of 20 gonococcal clinical isolates from patients with mucosal or disseminated gonococcal infections (35). Variable presentation of KDO on gonococcal LOS may be due to the absence of a terminal galactose acceptor if LgtA is switched off (Fig. 1) or via competition if another glycosyltransferase, LgtD, is phase varied “on” and transfers GalNAc in competition with Lst/CMP-KDO for the terminal galactose acceptor. The transfer of KDO to LOS was also investigated in N. meningitidis, which, unlike N. gonorrhoeae, can synthesize CMP-Neu5Ac via SiaB (27, 36). The ELISA results in Fig. 2C show the absence of KDO in the meningococcal strain MC58 wild type and *lst* mutant. However, an MC58 *siaB* mutant, which cannot synthesize CMP-Neu5Ac, was recognized by MAb 6E4, indicating the presence of KDO. These data indicate that there is competition between the CMP-Neu5Ac and CMP-KDO substrates for Lst-mediated transfer of the terminal sugar to LOS and that a mutation in N. meningitidis that ablates CMP-Neu5Ac synthesis is sufficient to permit KDO addition to LOS. To define the terminal KDO structure on the LOS, MAb 6E4 Western blotting with LOS purified from MC58, 1291, and their LOS glycotransferase (*lst* and *lgtB*) mutant strains was performed. LgtB is a β1,4-galactosyltransferase that transfers CMP-Neu5Ac and CMP-KDO receptor galactose (Gal) to the *N*-acetylglucosamine (GlcNAc). As shown in Fig. 1B, the purified LOS from the 1291 wild type and the MC58 *siaB* mutant was 6E4 positive, while the 1291 and MC58 *lst* and *lgtB* mutants all were 6E4 negative. LOS from the MC58 wild type was slightly 6E4 positive, indicating the presence of a small amount of KDO incorporated at the terminal on LOS.

### CMP-Neu5Ac competes with KDO for addition to N. gonorrhoeae LOS by Lst.

The gonococcus cannot synthesize CMP-Neu5Ac as a substrate for LOS sialylation (37) due to a lack of CMP-Neu5Ac synthetase. To determine whether the incremental addition of CMP-Neu5Ac to the growth media can compete with CMP-KDO for Lst addition to LOS and thereby reduced binding of MAb 6E4, N. gonorrhoeae strain 1291 was grown in 0, 30 μg/ml, and 100 μg/ml CMP-Neu5Ac and whole-cell ELISA was performed. The results in Fig. 2D show that dose-dependent inhibition of 6E4 MAb binding to whole cells begins at 30 μg/ml CMP-Neu5Ac and is increased at 100 μg/ml. These data suggest that CMP-Neu5Ac taken up from the media competes with endogenous CMP-KDO as a substrate for Lst activity.

### Lst is localized in the cytoplasm of pathogenic *Neisseria*.

**FIG 3:**
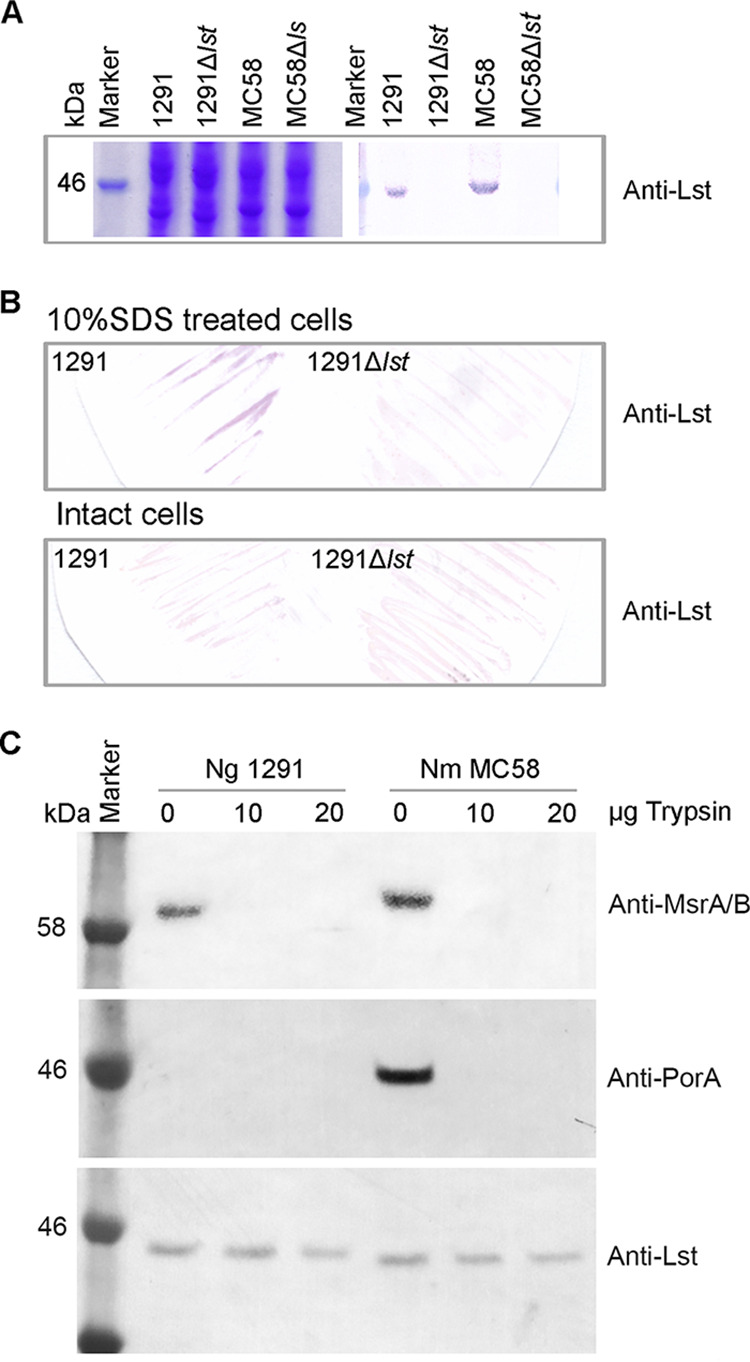
Cellular localization of Lst. (A) Western blot analysis of anti-Lst polyclonal antisera binding to Lst in whole-cell lysates. Coomassie staining gel showed equal loading of cell lysates. (B) Strain 1291 wild type and *lst* mutant were blotted on the membrane. Cells were treated with or without 10% SDS. Anti-Lst could detect Lst only when the cell was lysed. (C) Western blot analysis of whole, intact N. gonorrhoeae 1291 and N. meningitidis MC58 cells treated with increasing concentrations of trypsin (0, 10, and 20 μg) and probing with anti-Lst serum. Published neisserial MsrAB protein and the meningococcal PorA protein are the controls of surface protein.

The LOS core oligosaccharide is assembled on the inner face of the cytoplasmic membrane (38–40). The glycosyl transferases associated with LOS biosynthesis are localized in the cytoplasm, loosely associated with the membrane (reviewed in reference 38). Previously, the alpha-2,3-sialyltransferase Lst of N. gonorrhoeae was reported to be a surface-exposed outer membrane protein that could be a vaccine candidate (41). However, in the current study we demonstrate that KDO can be added to LOS in an Lst-dependent fashion. Furthermore, it is well established that CMP-KDO biosynthesis and utilization occur in the cytoplasm and that it has a very short half-life and a fast decomposition rate (42), indicating that Lst utilization of KDO must occur in the cytoplasm. Therefore, we performed a detailed investigation of the cellular location of Lst. First, SignalP (43) analysis predicts that Lst contains no N-terminal signal peptide, suggesting that it is not a secreted protein. Second, recombinant His-tagged Lst protein was expressed and purified and mouse polyclonal anti-Lst serum was produced. The anti-Lst serum bound specifically to the Lst protein in N. gonorrhoeae and N. meningitidis wild-type cell lysates in the Western blot analysis (Fig. 3A). Whole-cell colony blots revealed that the anti-Lst serum could only detect Lst when the cells were lysed by 10% SDS (Fig. 3B). Furthermore, trypsin digestion of surface-exposed proteins shows detection of Lst in whole-cell N. gonorrhoeae and N. meningitidis treated with 0, 10, and 20 μg of trypsin for 60 min (Fig. 3C). Lst was not digested by trypsin. However, the known outer membrane surface proteins MsrAB and PorA were both digested by the trypsin concentrations tested. CFU counts between pre- and post-trypsin treatment were measured to ensure trypsin digestion of the bacterial surface did not affect cell viability (data not shown). Together, these data indicate that Lst is located in the cytoplasm, while none of the data support localization of Lst on the surface of the outer membrane.

### The terminal KDO epitope is present in N. gonorrhoeae *in vivo*.

**FIG 4:**
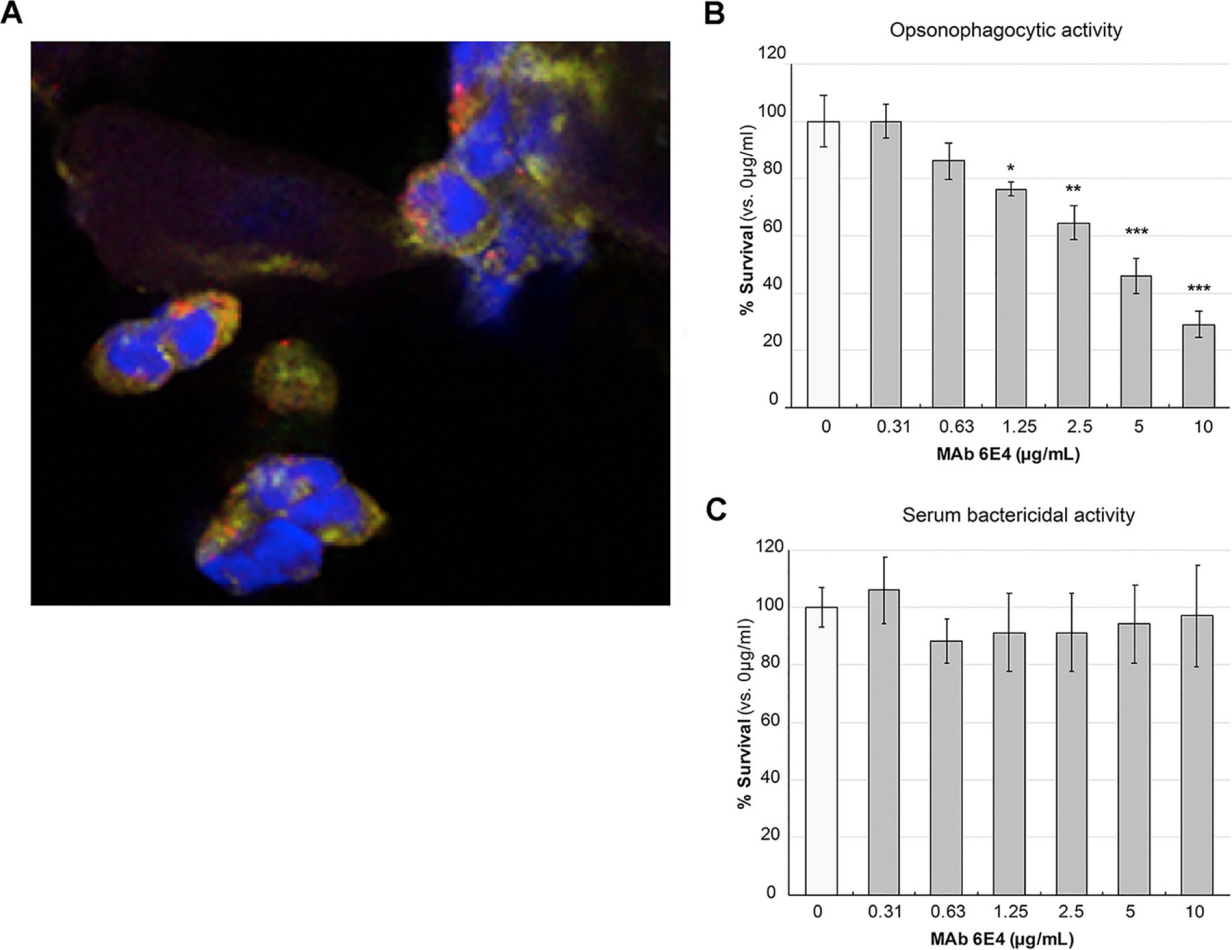
(A) Confocal microscopy study of cervical swab from a patient with a documented N. gonorrhoeae infection stained with both anti-KDO MAb 6E4 (Texas red) and anti-LOS MAb 6B4 (FITC). Infected PMNs can be seen with gonococci stained with both antibodies separately (red or green) and with antibodies colocalized to the same organisms (yellow). This study indicates that KDO termination occurs *in vivo* as well as *in vitro*. (B) Opsonophagocytic activity of MAb 6E4 against N. gonorrhoeae strain 1291. The survival of N. gonorrhoeae in the presence of 0 to 10 μg/ml MAb 6E4, primary human PMNs, and 10% normal human serum as a complement source is shown. Data represent the average survival (±1 standard deviation) for triplicate samples, shown as a percentage of bacteria in the absence of antibody (0 μg/ml MAb 6E4, set at 100%, represents 5.07 × 10^3^ CFU). There was a statistically significant difference between groups, as determined by one-way ANOVA [F(6,14) = 59.20, *P* < 0.0001]. Statistically significant differences relative to the untreated wild type, using a two-tailed Student's *t* test, are indicated: *, *P* < 0.05; **, *P* ≤ 0.01; ***, *P* ≤ 0.001. (*P* values were the following: 1.25 μg/ml, 0.012; 2.5 μg/ml, 0.005; 5 μg/ml, 0.001; and 10 μg/ml, 0.0003.) (C) Serum bactericidal activity. The survival of N. gonorrhoeae in the presence of 0 to 10 μg/ml MAb 6E4 and 10% normal human serum is shown. Data represent the mean survival (±1 standard deviation) for triplicate samples as a percentage of bacteria in the absence of antibody (0 μg MAb 6E4, set at 100%, represents 4.53 × 10^3^ CFU). There was no statistically significant difference between group means, as determined by one-way ANOVA [F(6,14) = 0.71, *P =* 0.65]. There was no statistically significant difference for any group relative to the untreated wild type, as determine using a two-tailed Student's *t* test (*P ≥ *0.12).

To examine if N. gonorrhoeae can incorporate KDO as the terminal glycan of LOS during *in vivo* infection, a confocal microscopy study was conducted using MAb 6E4 on the cervical swab from a patient with a documented N. gonorrhoeae infection. Figure 4A demonstrates that *in vivo*, the terminal KDO epitope is present in N. gonorrhoeae on cervical epithelial cells. This suggests that the free CMP-Neu5Ac in the cervical environment was not sufficient to inhibit Lst incorporating KDO into LOS.

### MAb 6E4 mediates opsonophagocytic killing.

KDO is a sugar that is not found in humans, and studies in H. influenzae have proposed KDO as a vaccine antigen (1). To examine if the surface-expressed KDO in N. gonorrhoeae could be a vaccine target, MAb 6E4 was used in opsonophagocytic killing and serum bactericidal assays against N. gonorrhoeae strain 1291. Dose-dependent opsonophagocytic killing was observed from 0.65 to 10 μg/ml MAb 6E4, with 5 μg/ml MAb 6E4 eliciting 54% killing and 10 μg/ml eliciting 71% killing relative to the no treatment control (Fig. 4B). No complement-dependent serum bactericidal activity was observed at concentrations up to 10 μg/ml MAb 6E4 (Fig. 4C). These data suggest that antibodies targeting KDO can mediate bacterial killing via opsonophagocytic activity and that the LOS terminal KDO epitope in N. gonorrhoeae is a potential vaccine target.

## DISCUSSION

N. gonorrhoeae is an obligate human pathogen that is highly adapted to the human environment and has numerous virulence factors that allow for the adherence, colonization, and avoidance of host complement responses. Gonococcal LOS is one of the important surface structures involved in pathogenesis. Due to its accessibility, gonococcal LOS has been proposed to be a vaccine candidate. In particular, the LOS epitope was recognized by MAb 2C7 (44). The MAb 6E4, used in this study, was previously generated by immunizing mice with NTHi LOS (45, 46), and the epitope recognized by 6E4 is KDO (29). KDO is a bacterium-specific carbohydrate that is common to the inner core of almost all Gram-negative bacteria, in which it plays an essential role linking lipid A to the oligo- or polysaccharide portion of LOS/lipopolysaccharide. Using the 6E4 MAb, we have made the unexpected discovery that the KDO epitope is also present on the LOS terminus in N. gonorrhoeae. Although the inner core KDO cannot be recognized by 6E4, we show that 6E4 detects a population of LOS molecules that have terminal KDO on LOS in the place where Neu5Ac is normally located. N. gonorrhoeae contains one LOS sialyltransferase, Lst, which is known to transfer Neu5Ac from CMP-Neu5Ac to the terminal end of LOS. However, here we have demonstrated that Lst can also use endogenous CMP-KDO as a substrate to transfer KDO to the LOS terminus in the absence of CMP-Neu5Ac.

**FIG 5:**
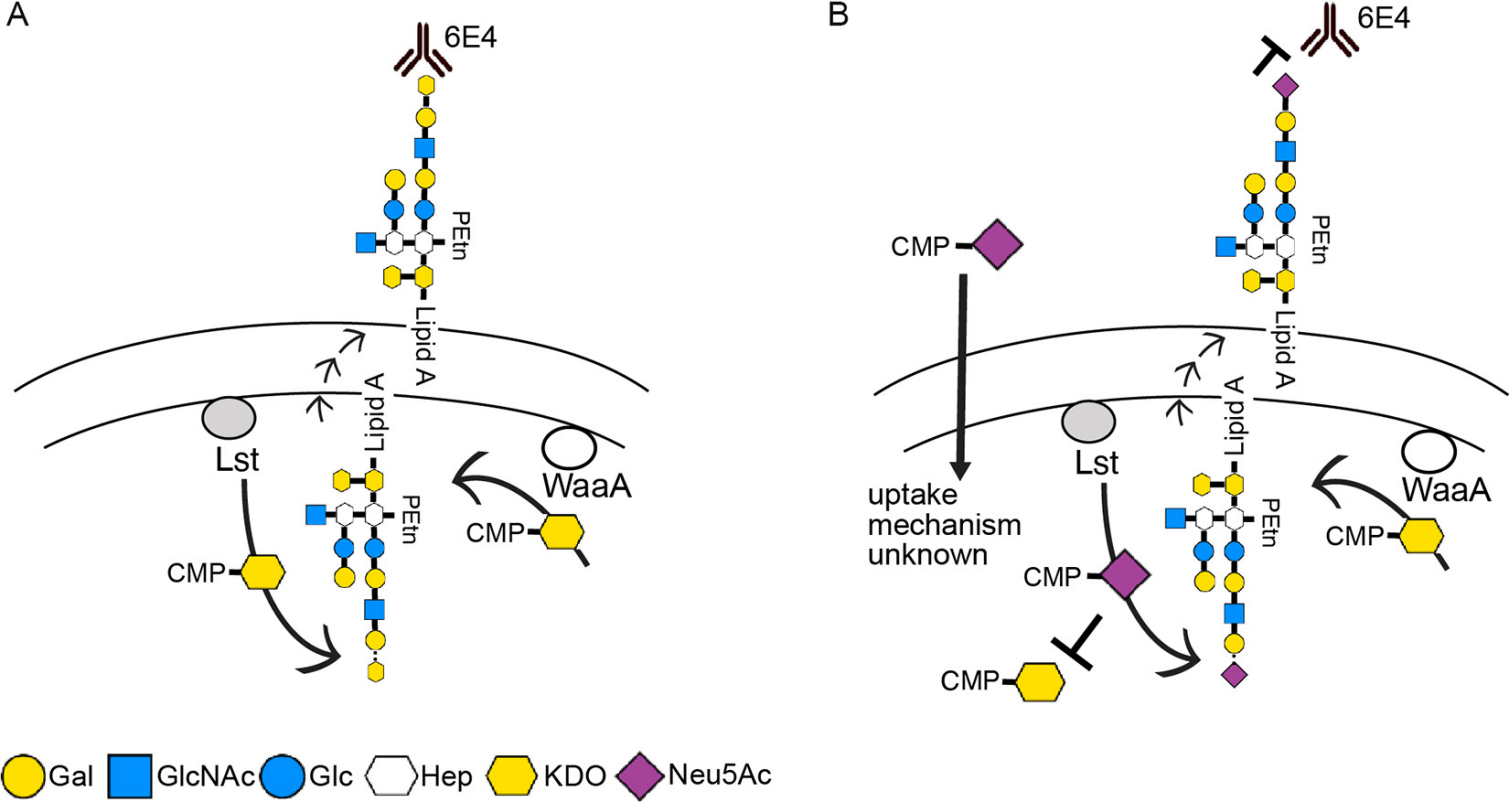
Schematic diagram of proposed biosynthesis pathway of terminal KDO on LOS in N. gonorrhoeae. (A) Inside cells, CMP-KDO is synthesized by WaaA and transferred to the terminal glycan of LOS by Lst in the absence of CMP-Neu5Ac. (B) In the presence of CMP-Neu5Ac, CMP-Neu5Ac can be taken up in the intracellular environment by an unknown mechanism and competes with CMP-KDO for addition to LOS by Lst inside cells.

The expression of KDO as a terminal modification of LOS has been reported in NTHi (29) and is dependent on the sialyltransferase LsgB. The expression of KDO as a terminal modification of LOS in the pathogenic *Neisseria* species is unexpected due to a study that reported that Lst is localized on the outer membrane (41). The earlier study used two experimental approaches to investigate localization, Western blot analysis of subcellular fractions of N. gonorrhoeae F62 and N. meningitidis MC58⊄3 with anti-Lst antiserum and binding of anti-Lst antiserum to whole-cell bacteria in an ELISA. In the cell fractionation and whole-cell binding assay studies, no control proteins (i.e., cytoplasmic, inner membrane, periplasmic, and outer membrane) were used to verify the identity or purity of fractionated material. Similarly, no control proteins were used to establish the status of the whole cells (i.e., intact or lysed) in ELISA. Thus, the conclusion that Lst in the pathogenic *Neisseria* is an outer membrane protein is not supported by strong evidence. Our study presents several lines of evidence that KDO is transferred to LOS by Lst in the cytoplasm. This is consistent with sialyltransferase localization in other Gram-negative pathogens: NTHi (29), Helicobacter acinonychis, and Helicobacter pylori (47). N. gonorrhoeae requires exogenous CMP-Neu5Ac to sialyate LOS and can take up CMP-Neu5Ac from growth media, suggesting a novel CMP-Neu5Ac transport system that delivers CMP-Neu5Ac to the cytoplasm, as shown in Fig. 5. Nucleotide sugar transporters have not been reported in bacterial systems but present eukaryotes to transport nucleotide sugars to the lumen of organelles that are the site of oligosaccharide biosynthesis (48).

Our studies with N. meningitidis MC58⊄3, which endogenously synthesizes CMP-Neu5Ac, indicate that Neu5Ac is the preferred substrate for Lst. When both CMP-Neu5Ac and CMP-KDO are present in wild-type MC58⊄3, only trace amounts of KDO are detectable on LOS, whereas when CMP-Neu5Ac biosynthesis is knocked out (SiaB mutant), KDO is readily detected (Fig. 1B and 2C). In the case of N. meningitidis group Y strain, 406Y (NRCC 4030), which expresses L3 immunotype LOS (like MC58⊄3), no KDO has been observed linked to the terminal galactose of LOS in nuclear magnetic resonance (NMR) structural analysis (49). Moreover, no terminal KDO was observed in previous mass spectrometry analyses of the N. gonorrhoeae strain 1291 LOS structure (17). These studies are consistent with the KDO-terminated LOS representing a very small proportion of all LOS molecules present in the outer membrane. This is consistent with our observation with N. meningitidis strain MC58⊄3 described above, that CMP-KDO is not the preferred Lst substrate, and suggests that the low level of terminal KDO-LOS is refractory to both NMR and mass spectrometry approaches. Notwithstanding this limitation, the case that KDO is present in the same position as Neu5Ac in LOS of these species is supported by strong evidence, such as (i) the biophysical studies that defined MAb 6E4’s epitope as KDO (29), (ii) the Lst dependence of KDO expression on LOS (Fig. 1B and 2A to D), (iii) the requirement for the terminal galactose of LOS for addition of KDO, as evidenced by loss of KDO in a mutant (LgtB) that lacks terminal galactose (Fig. 1B), and (iv) the suppression of KDO addition to LOS by the presence of endogenous CMP-Neu5Ac (N. meningitidis, wild type versus SiaB) (Fig. 2C) or exogenous CMP-Neu5Ac (N. gonorrhoeae) (Fig. 2D).

In this study, we show that KDO can be transferred to the terminal of LOS by Lst in the absence of Neu5Ac, and the majority of N. gonorrhoeae clinical isolates that we surveyed express KDO on their cell surface. This incorporation of KDO also occurs *in vivo*, with KDO detected on N. gonorrhoeae colonizing cervical epithelial cells in a cervical swab from a patient with a documented gonococcal infection. The host CMP-Neu5Ac level was not sufficient to suppress KDO incorporation. Significantly, our data showed that there is sufficient KDO-terminated LOS present on the bacterial surface such that MAb 6E4 can bind and kill N. gonorrhoeae in an opsonophagocytic killing assay, suggesting that terminal KDO on LOS is a vaccine candidate.

## MATERIALS AND METHODS

### Strains, plasmids, and culture conditions.

N. gonorrhoeae strain 1291, 1291*lst*, MS11, MS11*lst*, F62, and F62*lst* (50) were grown on GC agar (Oxoid) or GC broth with 1% IsoVitaleX, and N. meningitidis strain MC58, MC58*lst* (50), and MC58*siaB* (P. Chopra, J. Führing, P. Ng, T. Haselhorst, J. Dyason, F. Rose, R. Thomson, R. Gerardy-Schahn, D. Grice, M. Jennings, A. Münster-Kühnel, M. von Itzstein, submitted) were grown on 1% brain-heart infusion (BHI) agar and 10% Levinthal’s medium base at 37°C with 5% CO_2_. Twenty clinical isolates (35) of N. gonorrhoeae from disseminated or mucosal gonorrhoeae infection were also studied. In selected experiments, CMP-Neu5Ac at various concentrations was added to the medium as indicated.

### LOS purification.

Cells were grown on BHI or GC solid medium. Bacteria were collected by scraping and were suspended in a solution of 60 mM Tris, 10 mM EDTA, and 2% (wt/vol) sodium dodecyl sulfate, pH 6.8. Proteinase K was added to a final concentration of 50 μg/ml, and samples were incubated overnight at 37°C. Following three ethanol precipitations, samples were treated with DNase I and RNase A. Samples were then phenol extracted, ethanol precipitated three more times, and centrifuged at 120,000 × *g* for 75 min. The pellets were resuspended in water, frozen, and then lyophilized.

### Antibodies.

Monoclonal antibody (MAb) 6E4 is an IgG3 murine monoclonal antibody that recognizes a terminal KDO epitope (46). Polyclonal antisera, α-Lst, were produced by inoculating mice with purified recombinant Lst protein with Freund's adjuvant. The *lst* gene was amplified from MC58 using primers LstNcoI-F0or (5′-CTGCATCGTAGGCCATGGGCTTGAAAAAGGCTTG-3′) and LstNdeI Rev (5′-GCACTCGAGCATATGTCATTAGTGGTGATGGTGGTGATGATTTTTATCGTCAAATGTCAAAATCGGG-3′). The PCR products were cloned into vector pET-15b (Novagen), and the resulting plasmids were transformed into the Escherichia coli BL21 Star (DE3)pLysS host strain (Novagen) for overexpression. The expression and purification of Lst are the same as previously described for other neisserial LOS glycotransferases (51, 52).

### Whole-cell ELISA.

Bacteria were grown on BHI or GC plates for 16 h. Cells were harvested and resuspended in PBS at an optical density at 600 nm of 0.20. Microtiter plate wells were filled with 50 μl of the bacterial suspension and dried at room temperature overnight in the laminar flow cabinet. After the bacteria in the dried wells were heat killed for 1 h at 56°C, the wells were washed and ELISA was performed with MAb 6E4 at a dilution of 1:64. Secondary antibody (polyclonal anti-mouse Ig HRP; P044701; Dako) was used at a dilution of 1:2,000. The substrate TMB (3,3′,5,5-tetramethylbenzidine) solution (ThermoFisher Scientific) was used per the manufacturer’s instructions. Equal amounts of 1 N hydrochloric acid were added to stop the reaction. Absorbance was read in a Tecan model Infinite 200 Pro plate reader at 450 nm.

### Fluorescent labeling of clinical samples.

Paraformaldehyde-fixed archival clinical specimens from cervical swabs obtained from patients presenting with gonorrhea were dried onto glass microscope slides.

These samples were incubated simultaneously with MAbs 6E4 (IgG) and 6B4 (IgM). Fluorescent label was provided by incubation with goat anti-mouse IgG3/FITC to label MAb 6E4 plus goat anti-mouse IgM/Texas red for MAb 6B4 (both conjugates from Jackson ImmunoResearch). The samples were counterstained with Draq5 (Cell Signaling Technologies) to label cell nuclei and viewed by confocal microscopy on a Leica STED system in the Central Microscopy Core Facility at the University of Iowa.

### Human polymorphonuclear leukocyte (PMN) and serum collection.

Whole blood from healthy volunteers was collected by venipuncture with informed consent and the approval of the Griffith University Human Ethics Committee (HREC 2012/798). Blood was collected in K3 EDTA or Vacuette Z serum separator tubes (Greiner Bio-One) for PMN or serum preparation, respectively. PMNs were isolated using Polymorphprep (Axis-Shield) per the manufacturer’s instructions. For serum isolation, blood was allowed to clot for 15 min at room temperature and then centrifuged for 10 min at 2,000 × *g*.

### Opsonophagocytic killing and serum bactericidal assays.

A modified version of the opsonophagocytic assay (53) was used, where N. gonorrhoeae 1291 (∼1 × 10^3^ CFU) was incubated in serial dilutions of MAb 6E4 (0 to 10 μg) for 15 min at 37°C. PMNs (∼1 × 10^5^ cells) and 10% normal human serum as a complement source (preabsorbed with N. gonorrhoeae [54]) were then added and incubated at 37°C for 90 min. Serum bactericidal assays were performed by incubating N. gonorrhoeae 1291 (∼1 × 10^3^ CFU), MAb 6E4 (0 to 10 μg/ml), and 10% preabsorbed normal human serum for 30 min, as described previously (54). For both assays, gonococcal survival was determined after plating of serial dilutions on GC agar, and survival was calculated as a percentage relative to the no-antibody control. Statistical significance was calculated using one-way analysis of variance (ANOVA) and Student's *t* test.
